# Safety Net Primary Care Capabilities After the COVID-19 Pandemic

**DOI:** 10.1001/jamahealthforum.2024.2547

**Published:** 2024-08-16

**Authors:** Karen E. Schifferdecker, Ching-Wen W. Yang, Matthew B. Mackwood, Hector P. Rodriguez, Stephen M. Shortell, Ellesse-Roselee Akré, A. James O’Malley, Caryn Butler, Alena D. Berube, Alice O. Andrews, Elliott S. Fisher

**Affiliations:** 1 Dartmouth Institute for Health Policy and Clinical Practice, Geisel School of Medicine at Dartmouth, Lebanon, New Hampshire; 2 Department of Community and Family Medicine, Geisel School of Medicine at Dartmouth, Lebanon, New Hampshire; 3 Center for Healthcare Organizational and Innovation Research, University of California, Berkeley; 4 Johns Hopkins Bloomberg School of Public Health, Baltimore, Maryland; 5 Department of Biomedical Data Science, Geisel School of Medicine at Dartmouth, Lebanon, New Hampshire; 6 Department of Medicine, Geisel School of Medicine at Dartmouth, Lebanon, New Hampshire

## Abstract

**Importance:**

Federally qualified health centers (FQHCs) provide care to 30 million patients in the US and have shown better outcomes and processes than other practice types. Little is known about how the COVID-19 pandemic contributed to FQHC capabilities compared with other practices.

**Objective:**

To compare postpandemic operational characteristics and capabilities of FQHCs with non-FQHC safety net practices and non-FQHC, non–safety net practices.

**Design, Setting, and Participants:**

This nationally representative survey conducted from June 2022 to February 2023 with an oversampling of safety net practices in the US included practice leaders working in stratified random selection of practices based on FQHC status, Area Deprivation Index category, and ownership type per a health care network dataset.

**Exposures:**

Practice type: FQHC vs non-FQHC safety net and non-FQHC practices.

**Main Outcomes and Measures:**

Primary care capabilities, including 2 measures of access and 11 composite measures.

**Results:**

A total of 1245 practices (221 FQHC and 1024 non-FQHC) responded of 3498 practices sampled. FQHCs were more likely to be independently owned and have received COVID-19 funding. FQHCs and non-FQHC safety net practices were more likely to be in rural areas. FQHCs significantly outperformed non-FQHCs on several capabilities even after controlling for practice size and ownership, including behavioral health provision (mean score, 0.53; 95% CI, 0.51-0.56), culturally informed services (mean score, 0.55; 95% CI, 0.53-0.58), screening for social needs (mean score, 0.43; 95% CI, 0.39-0.47), social needs referrals (mean score, 0.53; 95% CI, 0.48-0.57), social needs referral follow-up (mean score, 0.31; 95% CI, 0.27-0.36), and shared decision-making and motivational interviewing training (mean score, 0.53; 95% CI, 0.51-0.56). No differences were found in behavioral and substance use screening, care processes for patients with complex and high levels of need, use of patient-reported outcome measures, decision aid use, or after-hours access. Across all practices, most of the examined capabilities showed room for improvement.

**Conclusions and Relevance:**

The results of this survey study suggest that FQHCs outperformed non-FQHC practices on important care processes while serving a patient population with lower incomes who are medically underserved compared with patients in other practice types. Legislation to expand funding for the FQHC program should improve services for underserved populations and target current non-FQHC safety net practices to serve these populations. Increased support for these practices could improve primary care for rural populations.

## Key Points

**Question:**

How was the COVID-19 pandemic associated with safety net practice capabilities, especially when comparing federally qualified health centers (FQHCs) and non-FQHC practices?

**Findings:**

In this nationally representative survey study of 1245 primary care practices, FQHCs outperformed non-FQHCs on capabilities for patient care. FQHC and non-FQHC safety net practices were more likely to be located in rural communities, and all practices underperformed on most of the capabilities examined.

**Meaning:**

The results of this study suggest the proposed policies to expand FQHCs may improve capabilities of primary care practices and should focus on practices serving safety net populations that are not yet FQHCs, which may lead to improved access to high-quality care, particularly for rural populations.

## Teaser

This survey study compares post–COVID-19 pandemic operational characteristics and capabilities of federally qualified health centers with non–federally qualified health centers.

## Introduction

Federally qualified health centers (FQHCs) play a vital role in delivering primary care services to 30 million individuals, particularly in areas with limited access to health care and socioeconomic opportunities.^1^ These centers, which are designed to meet the unique needs of underserved populations, receive enhanced federal funding to meet specific standards of care.^2^ Although the Affordable Care Act substantially expanded the number of FQHCs,^2^ new centers were less likely to be in rural areas or areas with high poverty levels.^3^ As such, many individuals who are uninsured, underinsured, or have Medicaid coverage are still served by non-FQHC practices. Earlier work has compared the characteristics and capabilities of FQHCs, non-FQHC practices serving populations with low incomes, and other practices and found that FQHCs performed better on many domains relevant to caring for populations with fewer advantages.^4^

The COVID-19 pandemic profoundly affected populations with fewer advantages, with an outsized effect on people living in areas with greater economic deprivation and among individuals of racial and ethnic minority groups.^5,6^ While some of the health effects were due to higher rates of comorbidities and risk of infection, these disparate outcomes exposed critical contributors to COVID-19 infection that were associated with low socioeconomic status and limited access to care.^6^ These associations were exacerbated by pandemic-related staffing shortages and burnout.^7^ Given the focus of FQHCs on populations with fewer advantages, that 63% of the patients that they serve are of racial and/or ethnic minority groups,^1^ and the still important role of non-FQHC practices serving as safety nets, it is critical to examine how safety net and non–safety net practices have fared as the pandemic has waned. Particularly important gaps in knowledge include the capabilities of safety net and non–safety net practices, how federal pandemic funds were distributed,^8^ and the current level of financial strain faced by primary care practices.

We conducted a national survey of primary care practices, oversampling FQHCs and non-FQHC safety net practices. The survey was conducted between June 2022 and February 2023, allowing insights into the characteristics and capabilities of safety net and non–safety net practices as the US emerged from the pandemic.

## Methods

We used a cross-sectional, nationally representative survey study design and followed the Consensus-Based Checklist for Reporting Survey Studies (CROSS).^9^ Dartmouth College’s Committee for the Protection of Human Subjects approved the study as exempt. The survey instrument included 52 items selected based on our prior studies^10,11^ of primary care practices and expanded with items drawn from the Culturally and Linguistically Appropriate Services standards.^12^ Study team members included primary care physicians and health services, health equity, and public health researchers.

Similar to the previous study,^4^ we used stratified-cluster sampling of primary care practices, excluding pediatric practices. The sample consisted of prior study respondent practices and a stratified random selection of additional practices based on FQHC status, area deprivation, and ownership type (independent, medical group, system) (Figure).

**Figure.**
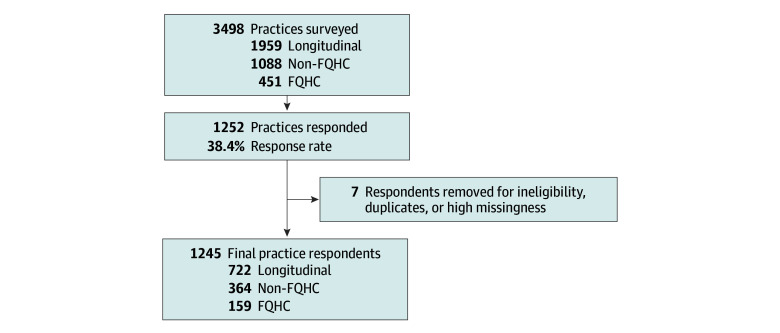
Flow of Sample and Respondent Practices Based on survey sample. FQHC indicates federally qualified health center.

SSRS, a market research firm, fielded the survey from June 2022 to February 2023 using 3 primary contacts per site who were practice managers or physician leaders. Outreach to each contact, done on a rolling basis until a response was received, consisted of a mailed notification, which was followed by a mailed survey packet with a $10 bill and a paid return envelope, and a second mailed survey packet with a paid return envelope. Packets included individualized links to an online survey alternative and a promised $40 check when the survey was received.

### Measures

As in previous work,^4^ we created 3 practice type categories. We defined FQHCs based on survey response and crosschecked with the Health Resources and Services Administration’s list of FQHC and FQHC look-alike sites^13^; 10 FQHC look-alikes were included as FQHCs. We categorized the remaining practices as non-FQHC safety net practices or non-FQHC practices based on whether the practice reported 20% or more of their annual income as coming from uninsured and/or Medicaid patients.

To examine capabilities, we created 11 composite scores following the method described by Fisher^11^ to account for differences in missingness patterns across items for each composite score. Items for each composite were based on theoretical relevance to each other and were further investigated with Cronbach α and an exploratory factor analysis (eTable 1 in Supplement 1). Composites included behavioral health provision, opioid treatment, culturally informed services, behavioral and substance use screening, screening for social needs, social needs referrals, social needs referral follow-up, care processes for patients with complex needs and a high level of need, patient-reported outcomes measures collection, shared decision-making and motivational interviewing training, and decision aid use.

### Statistical Analysis

All analyses were performed using Stata, version 18.0 (StataCorp), and survey weights to account for the probability that a practice was sampled from the sampling frame of eligible practices (based on the 2022 population of practices in the US) and whether the practice responded to our survey (to account for nonresponse, see eTable 2 in Supplement 1 for details). We used weighted χ^2^ tests per our marginal weights (eMethods in Supplement 1) to examine differences in practice characteristics and 2 access measures across the practice types. We used 1-way analysis of variance F-tests to examine the association of FQHC status with each capability score and partially account for multiple comparisons, then performed linear regressions to do pairwise comparisons of the non-FQHCs against FQHCs. The analysis of variance and linear regressions were adjusted for ownership and practice size to account for potential greater resources available in these practices. Statistical significance was set at *P *< .05.

## Results

Respondents included 1245 practices (221 FQHC and 1024 non-FQHC) of 3498 practices sampled (35.6%; Figure). Table 1 provides an overview of respondent practices overall and by practice type (see eTable 2 in Supplement 1 for comparison with nonrespondent practices). While there were modest differences in distribution across regions, FQHCs were more likely to be larger and independently owned. FQHCs and non-FQHC safety net practices were also more likely to be in rural areas.

**Table 1.** Characteristics of Participating Practices Overall and by Federally Qualified Health Center (FQHC) and Safety Net Status

Characteristic^a^	No. (weighted %)			
	Overall (N = 1245)	FQHC (n = 221)	Non-FQHC safety net (n = 237)	Non-FQHC non–safety net (n = 787)
Open longer weekdays	681 (56.6)	153 (68.3)	117 (51.3)	411 (55.5)
Open on weekends	376 (32.0)	93 (41.4)	63 (24.3)	220 (32.2)
Size of practice^b^^,^^c^				
Small (<5 physicians)	468 (40.1)	58 (27.4)	88 (49.8)	322 (39.9)
Medium (5-19 physicians)	611 (49.5)	134 (62.9)	101 (32.6)	376 (51.8)
Large (≥20 physicians)	151 (10.4)	22 (9.7)	47 (17.7)	82 (8.3)
Census regions^b^^,^^c^				
New England	100 (7.6)	23 (9.3)	21 (4.0)	56 (8.3)
Middle Atlantic	139 (14.0)	19 (8.1)	23 (14.3)	97 (15.2)
East North Central	202 (20.5)	32 (15.0)	36 (21.7)	134 (21.4)
West North Central	130 (11.0)	12 (5.7)	34 (17.9)	84 (10.1)
South Atlantic	214 (15.7)	31 (14.2)	35 (14.3)	148 (16.6)
East South Central	60 (4.8)	12 (5.4)	17 (7.3)	31 (4.0)
West South Central	94 (8.0)	14 (7.0)	22 (9.2)	58 (7.9)
Mountain	108 (6.8)	18 (6.6)	19 (5.7)	71 (7.3)
Pacific	198 (11.5)	60 (28.8)	30 (5.7)	108 (9.3)
Rural^b^^,^^c^	116 (7.5)	31 (13.5)	36 (10.3)	49 (5.4)
Ownership^c^^,^^d^				
Independently owned	515 (32.3)	91 (46.6)	77 (25.3)	347 (31.4)
A larger physician group	123 (8.0)	9 (4.8)	19 (6.2)	95 (9.2)
A hospital	123 (15.1)	3 (1.3)	30 (9.5)	90 (19.8)
A health care system	422 (40.9)	61 (23.4)	108 (58.0)	253 (39.5)
Other	55 (3.7)	50 (23.9)	3 (1.0)	2 (0.2)
Received federal COVID-19–related funding?^c^^,^^d^	820 (61.8)	184 (88.6)	136 (54.0)	500 (58.3)
Change in financial outlook before and after COVID-19^e^				
No change	702 (62.1)	127 (64.1)	141 (57.4)	434 (63.1)
Negative shift	419 (32.4)	57 (22.6)	82 (37.9)	280 (32.8)
Positive shift	81 (5.6)	27 (13.3)	12 (4.7)	42 (4.1)

^a^ Footnotes indicate the levels of significance for rejecting the null hypothesis that the relative frequency distribution of practice type (the columns) is the same across the levels of the given factor (the rows).

^b^ *P* < .01.

^c^ Size, census, and ownership are derived from IQVIA OneKey variables. Rurality is based on IQVIA zip code and assigned using Rural-Urban Commuting Area. COVID-19 funding was from the National Survey of Healthcare Organizations and Systems survey.

^d^ *P* < .001.

^e^ *P < *.05.

When examining potential COVID-19 financial association, FQHCs were substantially more likely to have received COVID-19 relief funding (184 [89%] vs 136 [54%] to 500 [58%]; *P* < .001) and appeared to have weathered the effect of COVID-19 financially better than non-FQHCs. Only 57 FQHCs (23%) reported worsened financial status compared with 82 (38%) and 280 (33%), respectively, for non-FQHC safety net practices and non-FQHCs.

When examining capabilities across practice types, FQHCs outperformed both types of non-FQHCs on 6 domains (Table 2): screening for social needs, social need referrals, social needs referral follow-up, culturally informed services, shared decision-making and motivational interview training, and behavioral health provision. In models that adjusted for practice size and ownership (eTable 3 in Supplement 1), these remained significant for FQHCs, as was opioid treatment. Aside from behavioral and substance use screening and, for FQHCs, screening for social needs and social needs referrals, most of the examined capabilities showed substantial room for improvement across practice types (Table 2).

**Table 2.** Comparison of Practice Capabilities by Federally Qualified Health Center (FQHC) Status

Characteristic	Mean score (95% CI)			
	Overall (N = 1245)	FQHC (n = 221)	Non-FQHC	
			Safety net (n = 237)	Non–safety net (n = 787)
Screening for social needs^a^	0.43 (0.39-0.47)	0.68 (0.63-0.72)	0.45 (0.38-0.52)	0.37 (0.32-0.42)
Social needs referrals^a^	0.53 (0.48-0.57)	0.78 (0.73-0.82)	0.51 (0.40-0.62)	0.47 (0.41-0.53)
Social needs referral follow-up^a^	0.31 (0.27-0.36)	0.46 (0.40-0.53)	0.27 (0.17-0.38)	0.29 (0.23-0.35)
Culturally informed services^a^	0.55 (0.53-0.58)	0.65 (0.62-0.68)	0.60 (0.55-0.65)	0.52 (0.49-0.55)
Care processes for patients with complex needs and high level of need	0.62 (0.60-0.65)	0.59 (0.56-0.63)	0.62 (0.57-0.67)	0.63 (0.60-0.67)
Shared decision-making and motivational interviewing training^a^	0.39 (0.36-0.43)	0.49 (0.45-0.53)	0.49 (0.41-0.56)	0.34 (0.30-0.38)
Decision aid use	0.43 (0.40-0.47)	0.39 (0.35-0.44)	0.52 (0.42-0.62	0.42 (0.37-0.46)
Behavioral health provision^a^	0.53 (0.51-0.56)	0.61 (0.58-0.64)	0.54 (0.48-0.60)	0.51 (0.48-0.54)
Behavioral and substance use screening	0.78 (0.75-0.80)	0.81 (0.78-0.83)	0.76 (0.70-0.83)	0.77 (0.74-0.81)
Opioid treatment	0.42 (0.39-0.44)	0.46 (0.43-0.49)	0.41 (0.35-0.48)	0.41 (0.38-0.43)
Patient-reported outcome measures	0.69 (0.66-0.72)	0.68 (0.64-0.72)	0.69 (0.60-0.77)	0.69 (0.65-0.74)

^a^ *P* < .001.

## Discussion

In this survey study, FQHCs outperformed non-FQHC practices, including non-FQHC safety net practices, on important capabilities, such as screening for social needs and behavioral health provision. This suggests that while the pandemic was challenging for all types of practices, FQHCs maintained capabilities that were important for meeting the needs of socioeconomically vulnerable patients who are often members of racial and ethnic minority groups, have lower incomes, and are medically underserved.

The equal or higher level of capabilities for FQHCs and their stronger financial status is likely due to the care standards required of FQHCs for federal funding and other streams of funding specifically for FQHCs, including COVID-19 relief funds.^14^ These findings suggest that FQHC expansion, supported by a recent Congressional Budget Office review of such proposed legislation,^15^ may be associated with improved access to practices better equipped to meet the needs of populations with fewer advantages, especially if expansion happened in rural communities. Nevertheless, our results show room for improvement on most capabilities across all practice types.

### Limitations

The modest response rate of this survey was similar to other large-scale surveys during the pandemic.^16^ Capabilities were self-reported and were not able to be independently verified.

## Conclusions

The results of this survey study suggest that expanding funding for FQHCs and improving the capabilities of non-FQHC safety net practices may be associated with improved access to high-quality primary care. Efforts to target practices serving safety net populations that are not now FQHCs deserves serious consideration for funding and may be associated with improved care for rural populations.

## Notes

Supplement 1. eTable 1. Full Survey Questions for Access and Composite Measures eTable 2. Comparison of NSHOS II Respondent and Non-Respondent Characteristics eMethods. Survey Non-response Weights Description eTable 3. Linear Regression for Comparison of Practice Capabilities Scores Between FQHCs and non-FQHCs

Supplement 2. Data sharing statement
