# Supplementary material for: Safety Net Primary Care Capabilities After the COVID-19 Pandemic
Source: JAMA Health Forum. 2024 Aug 16;5(8):e242547. doi: 10.1001/jamahealthforum.2024.2547 (PMC11329874; doi:10.1001/jamahealthforum.2024.2547)
Supplement: Supplement 1. — eTable 1. Full Survey Questions for Access and Composite Measures eTable 2. Comparison of NSHOS II Respondent and Non-Respondent Characteristics eMethods. Survey Non-response Weights Description eTable 3. Linear Regression for Comparison of Practice Capabilities Scores Between FQHCs and non-FQHCs [file jamahealthforum-e242547-s001.pdf]

Supplemental Online Content

Schifferdecker KE, Yang C-W W, Mackwood MB, et al. Safety net primary care capabilities after the COVID-19 pandemic. *JAMA Health Forum*. Published online August 16, 2024. doi:10.1001/jamahealthforum.2024.2547

eTable 1. Full Survey Questions for Access and Composite Measures
eTable 2. Comparison of NSHOS II Respondent and Non-Respondent Characteristics
eMethods. Survey Non-response Weights Description
eTable 3. Linear Regression for Comparison of Practice Capabilities Scores Between FQHCs and non-FQHCs

This supplemental material has been provided by the authors to give readers additional information about their work.

© 2024 Schifferdecker KE et al. *JAMA Health Forum.*

eTable 1. Full survey questions for Access and Composite Measures

Access Questions and Composite Score Questions	*Requested Response*	*Items in Scale*	*Crohnbach's alpha (using standardized items)*
**Access**<br>Is your practice regularly open for patient appointments on… (do not include urgent care provided in a different location): Weekdays before 8am or after 5pm?	*Yes, No*	***1***	*NA*
**Access**<br>Is your practice regularly open for patient appointments on… (do not include urgent care provided in a different location): Weekends?	*Yes, No*	***1***	*NA*
**Behavioral health provision**<br>Do you have behavioral health services formally integrated into your practice?<br><br>How difficult is it for your practice to obtain: Behavioral health services for patients?	*Yes, No*<br><br>*Very difficult, Somewhat difficult, Not at all difficult*	***4***	*0.49*
**Opioid treatment**<br>Do any of the clinicians at your practice provide drug treatment (e.g., buprenorphine) for opioid use disorders?<br><br>How difficult is it for your practice to obtain: Treatment for patients w/ opioid use disorders?	*Yes, No*<br><br>*Very difficult, Somewhat difficult, Not at all difficult*		*0.55*
**Culturally informed services**<br>How often is your practice able to provide professional language assistance services for patients with limited English proficiency?<br><br>To what extent are clinicians in your practice representative of the race and ethnicity of the patients you serve?<br><br>Does your practice routinely collect information about patients':<br>Race and ethnicity<br>Preferred language<br><br>Does your practice require training for clinicians and staff on the following? Please respond for each item for clinicians and for staff.<br>Providing high quality care to socio-economically disadvantaged populations<br>Providing high quality care to immigrant populations<br>Providing high quality care to racially and ethnically diverse populations	*Never, Rarely, Occasionally, Often, Always*<br><br>*Not at all, A little, Some, Quite a lot*<br><br>*Yes, No*<br><br>*Clinicians: Yes, No*<br>*Staff: Yes, No*	***7***	*0.86*

How confident are you in your practice's ability to address issues of race, racism, and/or discrimination when they occur for:<br>Patients<br>Clinicians<br>Staff<br><br>Does your practice review its performance on delivery of preventive services stratified by the following patient characteristics?<br>Race and ethnicity<br>Preferred language<br>Sex<br>Payer status or payer type (e.g. Medicaid)	*Not at all, A little, Some, Quite a lot*<br><br><br><br><br>*Yes, at practice level;*<br>*Yes, at system level;*<br>*No*		
**Behavioral and substance use screening**<br>Does your practice have a system in place to routinely screen patients for:<br>Tobacco use<br>Opioid use specifically<br>Substance use disorders (other than tobacco and opioid)<br>Polypharmacy<br>Depression	*Yes, No*	***5***	*0.60*
**Screening for social needs**<br>Does your practice have a system in place to routinely screen patients for:<br>Low health literacy<br>Food insecurity<br>Housing instability<br>Utility needs<br>Interpersonal violence<br>Transportation needs<br>Need for financial assistance with medical bills<br>Medicaid eligibility<br>Adverse childhood experiences (ACES)	*Yes, No*	***9***	*0.89*
**Social needs referrals**<br>Does your practice have a system in place to make it easy for clinicians to refer patients for:<br>Food insecurity<br>Housing instability<br>Utility needs<br>Interpersonal violence<br>Transportation needs<br>Financial assistance<br>Home and community-based services	*Yes, No*	***7***	*0.92*
**Social needs referral follow-up**<br>Does your practice have a system in place to ensure patients follow through on referrals for:<br>Food<br>Housing<br>Interpersonal violence	*Yes, No*	***3***	*0.92*

© 2024 Schifferdecker KE et al. *JAMA Health Forum.*

**Care processes for complex, high-need patients**<br>Does your practice have a system in place to identify complex, high need patients?<br><br>For your complex, high need patients, how often is a non-physician in the practice involved in:<br>Helping the patient coordinate care across clinicians<br>Helping the patient adhere to the care plan<br>Supporting health risk modification<br>Supporting medication adherence	*Yes, No*<br><br>*Never, sometimes, often, always*	***5***	*0.90*
**Patient-reported outcome measures**<br>Does your practice collect patient-reported measures of:<br>Depression<br>Physical function or disability for older adult patients<br>Pain for diabetic patients<br>Pain for musculoskeletal hip, knee or back patients	*Yes, No*	***4***	*0.75*
**Shared decision-making and/or motivational**<br>Does your practice have clinicians/staff who are formally trained in motivational interviewing?<br><br>Considering the clinicians in your practice, how many: Are formally trained in shared decision-making	*Yes, clinicians only; Yes, staff only; Yes, both clinicians and staff; No*<br><br>*None, some, most, all*	***2***	*0.49*
**Decision aid use**<br>Considering the clinicians in your practice, how many:<br>Routinely use decision aids for breast cancer screening<br>Routinely use decision aids for initial treatment for diabetes<br>Routinely use decision aids for knee joint replacement	*None, some, most, all*	***3***	*0.89*

© 2024 Schifferdecker KE et al. *JAMA Health Forum.*

eTable 2. Comparison of NSHOS II Respondent and Non-Respondent Characteristics

	Respondents (n=1,311)	Non-Respondents (n=2,187)	Sample Frame* (n=11,364)
**Size**			
% Solo: 1 Physician (n)	2.95 (38)	2.57 (55)	2.38 (257)
% Small: 2-9 Physicians (n)	69.77 (900)	65.47 (1,399)	67.56 (7,285)
% Medium: 10-20 Physicians (n)	15.74 (203)	17.31 (370)	16.95 (1,828)
% Large: 21+ Physicians (n)	11.55 (149)	14.65 (313)	13.10 (1,41)
Mean # of Physicians† (SD)	10.85 (21.68)	13.55 (69.83)	12.31 (38.58)
Mean # of Primary Care Physicians (SD)	7.04 (8.08)	8.09 (16.05)	6.95 (11.65)
Mean # of Specialists (SD)	3.80 (16.23)	5.45 (56.43)	5.37 (30.2)
Mean # of Advanced Practice Clinicians (SD)	4.46 (6.57)	4.93 (13.43)	4.53 (8.8)
**Geography**			
% Metropolitan (n)	80.40 (960)	85.03 (1,687)	83.21 (8,956)
% Micropolitan (n)	10.64 (127)	7.96 (158)	8.78 (945)
% Small Town (n)	6.37 (76)	5.24 (104)	5.47 (589)
% Rural (n)	2.60 (31)	1.76 (35)	2.54 (273)
% Northeast (n)	19.72 (255)	19.81 (426)	19.36 (2,095)
% Midwest (n)	26.30 (340)	26.23 (564)	28.97 (3,135)
% South (n)	29.54 (382)	30.42 (654)	29.85 (3,230)
% West (n)	24.44 (316)	23.53 (506)	21.81 (2,360)
**Practice Ownership**			
% Independent (n)	24.15 (312)	15.73 (338)	15.53 (1,679)
% Medical Group (n)	31.35 (405)	34.53 (742)	22.02 (2,380)
% Hospital/ health care system (n)	44.50 (575)	49.74 (1,069)	62.45 (6,750)

*Includes surveyed and non-surveyed organizations. †Physicians = All MDs/DOs; Sum of Primary Care and Specialist Physicians within practice.

© 2024 Schifferdecker KE et al. *JAMA Health Forum.*

eMethods. Survey Non-response Weights Description

Our sample consisted of prior study respondent practices and a stratified random selection of additional practices based on FQHC status, area deprivation, and ownership type (independent, medical group, system). Analyses were performed using survey weights to account for the probability that a practice was part of the original, prior study sample or was sampled from the sampling frame of eligible practices (established based on the 2022 population of medical practices in the US), and whether the practice responded to our survey (to account for non-response). Because practices that were part of the prior study were retained with probability 1, their effective sampling probability was 1 with respect to the 2022 sampling frame for this study. For newly eligible practices, we included 100% of FQHCs and an almost equal number (166 or 167) of non-FQHCs (1000 total) for the six combinations of system-type and the binary variable indicating whether the Federal Information Processing Standards (FIPS) decile was ^{3} 8. Thus, for our entire study sample, there were seven (six non-FQHCs and FQHCs) types of practices in total. For the newly eligible study sample, the sampling probabilities were simple proportions equal to the numerator divided by the number of practices in the original 2022 sampling frame (prior to knowing information regarding eligibility that was learned through the survey process) of the seven types of practices. Upon learning more about the practices after sampling them, some of the sampled practices were deemed to be ineligible, yielding a seven proportions of eligible practices. Of the sampled practices that were eligible, a certain fraction responded, yielding seven proportions of responders. Therefore, the joint probability of a practice being sampled, found to be eligible, and responding was estimated as the product of the corresponding three proportions for each of the seven types of practices. The inverses of these joint probabilities formed the marginal weights used in our chi-square test analyses.

© 2024 Schifferdecker KE et al. *JAMA Health Forum.*

eTable 3: Linear Regression for Comparison of Practice Capabilities Scores between FQHCs and non-FQHCs

	Behavioral health provision	Opioid treatment	Cuturally informed Services	Behavioral and substance use screening	Screening for social needs	Social needs referrals	Social needs referral follow-up	Care process for complex, high-needs patients	Patient-reported outcome measures	Shared decision-making and/or motivational interviewing training	Decision aid use
**ANOVA Type III for FQHC status**	F: 10.88***	F: 3.24*	F: 21.68***	F: 0.60	F: 39.22***	F: 30.86***	F: 8.70***	F: 0.44	F: 0.04	F: 11.60***	F: 1.96
**Independent Variable: FQHC status**											
FQHC	(base)	(base)	(base)	(base)	(base)	(base)	(base)	(base)	(base)	(base)	(base)
Non-FQHC with safety net	-0.086*	-0.057	-0.094**	-0.030	-0.242***	-0.280***	-0.206**	0.019	-0.014	0.004	0.110
	(0.036)	(0.039)	(0.033)	(0.037)	(0.047)	(0.062)	(0.066)	(0.034)	(0.050)	(0.048)	(0.058)
Non-FQHC w/o safety net	-0.108***	-0.059*	-0.156***	-0.025	-0.327***	-0.324***	-0.198***	0.027	-0.001	-0.134***	0.012
	(0.023)	(0.024)	(0.024)	(0.024)	(0.037)	(0.043)	(0.051)	(0.029)	(0.033)	(0.031)	(0.038)

ANOVA Type III F-statistic and associated p-value reported to account for multiple testing. Regression models are adjusted for size of practice (based on number of physicians) and ownership category. Asterisks indicate the levels of significance, *=p<.05, ** = p<0.01, *** = p<0.001.

© 2024 Schifferdecker KE et al. *JAMA Health Forum.*
