# Supplementary material for: Safety Net Primary Care Capabilities After the COVID-19 Pandemic
Source: JAMA Health Forum. 2024 Aug 16;5(8):e242547. doi: 10.1001/jamahealthforum.2024.2547 (PMC11329874; doi:10.1001/jamahealthforum.2024.2547)
Supplement: Supplement 2. — Data sharing statement [file jamahealthforum-e242547-s002.pdf]

Data Sharing Statement

Schifferdecker. Safety Net Primary Care Capabilities After the COVID-19 Pandemic. *JAMA Health Forum*. Published August 16, 2024. doi:10.1001/jamahealthforum.2024.2547

Data
Data available: Yes
Data types: Deidentified participant data, Data dictionary
How to access data: karen.e.schifferdecker@dartmouth.edu
When available: With publication

Supporting Documents
Document types: Other (please specify)
Additional Information: Survey instrument
How to access documents: karen.e.schifferdecker@dartmouth.edu
When available: With publication

Additional Information
Who can access the data: researchers whose proposed use of the data has been approved
Types of analyses: for any purpose
Mechanisms of data availability: after approval of a proposal
